# Student COVID-19 vaccination preferences in China: A discrete choice experiment

**DOI:** 10.3389/fpubh.2022.997900

**Published:** 2022-10-19

**Authors:** Siyuan Wang, Elizabeth Maitland, Tiantian Wang, Stephen Nicholas, Anli Leng

**Affiliations:** ^1^The George Institute for Global Health, UNSW Sydney, Newtown, NSW, Australia; ^2^School of Management, University of Liverpool, Liverpool, United Kingdom; ^3^Qingdao Municipal Center for Disease Control & Prevention, Qingdao, China; ^4^Australian National Institute of Management and Commerce, Sydney, NSW, Australia; ^5^Newcastle Business School, University of Newcastle, Newcastle, NSW, Australia; ^6^School of Political Science and Public Administration, Shandong University, Qingdao, China; ^7^Center for Health Preferences Research, Shandong University, Jinan, China

**Keywords:** student vaccination, COVID-19 vaccination, vaccination preference, DCE, vaccination utility

## Abstract

**Objective:**

This study uses a discrete choice experiment (DCE) questionnaire to investigate student vaccination preferences for both intrinsic and extrinsic attributes.

**Methods:**

A two-part DCE questionnaire was distributed to 1,138 students through face-to-face interviews at vaccination centers in Qingdao, China. Conditional logit models were used to understand student preference trade-offs. Mixed logit models (MLM) and sub-group analysis were conducted to understanding student preference heterogeneity.

**Results:**

We found that students preferred vaccines with fewer side effects (β = 0.845; 95% CI, 0.779–0.911), administered through third level health facilities (β = 0.170; 95% CI, 0.110–0.230), and had at least 1 year duration of protection (β = 0.396; 95% CI, 0.332–0.461. Higher perception of COVID-19 risks (β = 0.492; 95% CI, 0.432–0.552) increased the likelihood of student vaccination uptake. Surprisingly, vaccine effectiveness (60%) and percentages of acquaintances vaccinated (60%) reduced vaccination utility, which points to free-rider problems. In addition, we find that student study majors did not contribute to preference heterogeneity, and the main disparities in preferences were attributed to student risk tolerances.

**Conclusion:**

Both intrinsic and extrinsic attributes were influential factors shaping student preferences for COVID-19 vaccines. Our results inform universities and local governments across China on targeting their vaccination programs.

## Introduction

Between January and June of 2022, China experienced local COVID-19 outbreaks across Xian, Changchun, Beijing, Qingdao, Shanghai and other cities, resulting in partial or full regional-wide lockdowns. For example, one of the hardest hit cities, Shanghai recorded over 20 thousand daily infections and over 50 daily deaths during the peak of its outbreak (1). To control regional Covid outbreaks, the government implemented prevention and control measures, including lockdowns, school and university closures, routine testing and mandatory face masks in public (2). Vaccinations, as part of the government's COVID-19 measures (2), play a key role in preventing and controlling the spread of the Omicron variant. In December 2020, the Chinese government decreed a two-dose free and voluntary vaccination program for all eligible citizens (3). Inactivated vaccines account for the majority market share for general usage, with vaccine efficacy of 72.8% (3), and an acceptable safety record (4). While much of the current vaccination efforts are targeting the low vaccination rates among the elderly, young people are increasingly becoming the primary risk drivers of COVID-19 transmissions (5), with college students being a particularly high-risk cohort. Colleges have the same free and voluntary vaccination policy, but have relatively higher vaccination uptake than the general population due to targeted educational campaigns and herd behaviors. However, universities are still prone to outbreaks, for example, Jilin's 2-month provincial-wide lockdown was triggered by an initial college outbreak in Changchun, Jilin's provincial capital. As universities resume on-campus teaching, the need to understand the preferences and barriers to student vaccination is an increasingly important public health issue.

Previous studies have used various methods to investigate the vaccine acceptance, vaccine willingness and vaccine hesitancy of the general public (6–9). Misinformation, lack of trust, vaccination costs, perceptions of vaccine benefits and risks, concerns for vaccine efficacy and safety, and socio-demographic characteristics were reported as contributors to the public's COVID-19 vaccine uptake (10–14).

Discrete choice experiments (DCEs) are a quantitative method to examine stated preferences over hypothetical alternative scenarios (15). Widely used to reveal the preference trade-offs for vaccine related-characteristics, social characteristics and cognition both before and after the implementation of China's 2021 COVID-19 vaccination program (16–18), the theoretical basis of the discrete choice model is the theory of consumer demand choice, where consumers fully understand their preferences, and random utility theory, where consumers can consistently order rank their choices based on their preferences (19). In DCEs, respondents are asked to select from different scenarios comprising hypothetical alternatives to maximize their utility. Each scenario has a number of attributes (vaccine effectiveness, side-effects, vaccination sites, protection duration, acquaintances vaccinated and risk factors) and each attribute has different levels, as shown in Table 1. DCEs quantify the change in utility for alternative attribute levels, providing a superior framework for understanding preference trade-offs (20). In contrast, most mainland Chinese students' COVID-19 vaccination studies, encompassing vaccination acceptance, willingness and hesitancy, mainly used non-DCE online surveys (21–25), reporting similar vaccine hesitancy factors (21–25). In terms of student vaccine heterogeneity, the existing literature suggests that student majors do not contribute to differing vaccination preferences (26), except for medical or nursing students who exhibit overall higher vaccination willingness (27, 28). Two DCE studies identified efficacy, safety, number of doses, origin of vaccine and costs in student vaccination preferences, but for students in Hong Kong (29, 30).

**Table 1:** Attributes and levels used in the discrete choice experiment.

**Attributes**	**Levels**	**Descriptions**
Vaccine effectiveness	40%	Protects 40% of vaccinated
	60%	Protects 60% of vaccinated
	85%	Protects 85% of vaccinated
Self-assessed vaccine-related side effects	50/100,000	50 out of 100,000 risk of severe side effects
	10/100,000	10 out of 100,000 risk of severe side effects
	1/100,000	1 out of 100,000 risk of severe side effects
Vaccination sites	Level 1	Village clinic or community health station
	Level 2	Township or community health center
	Level 3	County hospital and above
Duration of vaccine protection	6 months	Six months of vaccine protection
	1 year	1 year of vaccine protection
	More than 2 years	More than 2 years
Acquaintances vaccinated	30%	30% of your family, friends and acquaintances already vaccinated
	60%	60% of your family, friends and acquaintances already vaccinated
	90%	90% of your family, friends and acquaintances already vaccinated
Risk perception (probability yourself and acquaintances being infected with COVID-19)	100/100,000	100 out of 100,000 contracting COVID-19
	6/100,000	6 out of 100,000 contracting COVID-19
	1/100,000	1 out of 100,000 contracting COVID-19

Our understanding of the vaccination preferences of Chinese university students remains contested. To address this lacuna, we conducted a face-to-face DCE investigation to assess the preference trade-offs for COVID-19 vaccinations of university students in Qingdao, a major economic, transport and education hub in coastal Shandong province, and home to over 360 thousand undergraduate students across more than 13 universities (31). Importantly, Qingdao was one of the first regions to roll out the COVID-19 vaccines, and one of the first to experience the 2022 COVID-19 outbreaks (32, 33). Importantly, our DCE model, extends many existing DCE frameworks of vaccine intrinsic attributes (effectiveness, vaccine-related side effects, protection duration and vaccination sites) by including extrinsic social relationship attributes (measured by the percentage of acquaintances vaccinated) and cognition factors (measured by the perception of risk) (17, 18). We also innovatively conducted sub-group analysis to investigate preference heterogeneity based on student majors and risk tolerance. We compare our results against previous DCE non-student and general public DCE studies to gain insight into any different barriers and motivations between student vaccination and general public preferences (16–18). The results of our study will inform universities and local governments on targeted vaccination policies for Chinese college students.

## Materials and methods

### Identification of attributes and levels

We adopted the well-developed framework from previous DCE COVID-19 studies as the basis for attribute and level setting (17, 18). As shown in Table 1, we identified six key attributes, comprising four vaccine intrinsic attributes (vaccine effectiveness, vaccine-related side effects, vaccination sties and duration of protection), and two vaccine extrinsic attributes, social relationships (percentage of acquaintances vaccinated) and cognition (risk perception) (17, 18). Vaccine effectiveness included 3 levels, 40, 60, and 85% and vaccine related side-effects consisted of 1/100,000, 10/100,000 and 50/100,000. Vaccination sites are set as three levels: village community health station (level 1), township community health station (level 2) and county hospitals (level 3). Duration of protection was divided into 6 months protection, 1 year protection and more than 2 years protection. Percentage of acquaintances vaccinated had three levels: 30% of your family, friends and acquaintances already vaccinated, 60% of your family, friends and acquaintances already vaccinated and 90% of your family, friends and acquaintances already vaccinated. Risk perception included 1/100000 chance of contacting COVID-19, 6/100,000 probability of contacting COVID-19 and 100/100,000 probability of contacting COVID-19. Details of attribute levels are shown in Table 1. In addition, other individual-specific characteristics, such as age, sex, urban-rural location and household income, trust in government and risk tolerance, were collected to investigate preference heterogeneity. Our DCE framework is consistent with the DCE COVID-19, seasonal influenza, H1N1 and Hepatitis B vaccine literature (17).

### Experimental design

A proven two-part questionnaire (18) was used to collect the data. Part one of the questionnaire obtained background characteristics, including sex (male, female), location (urban or rural based on household registration), family relationships (living with the elderly and children), academic major (humanities/social sciences or non-humanities/social sciences), risk tolerance (risk adverse, risk neutral and risk tolerant), and trust toward government (trust, unknown and mistrust). Part one also included a five-point Likert scale, where six questions surveyed each student's COVID-19 vaccination motives. Part two of the survey investigated student COVID-19 vaccination preference trade-offs. Consistent with Wang et al. (18) and Leng et al. (17), a D-efficient partial profile design was implemented for the main attributes and levels. Twenty-four hypothetical choice tasks were created and divided into three versions, each containing eight binary choice tasks. An additional choice task was added to each version, and later redacted from the analysis, to control for potential survey bias. An example of the choice task is given in Table 2.

**Table 2:** Choice set.

**Q1**	**Vaccine A**	**Vaccine B**
Vaccine effectiveness	40%	60%
Vaccine-related side effects	10/100,000	50/100,000
Vaccination sites	Level 1	Level 2
Duration of vaccine protection	1 year	6 months
Acquaintances vaccinated	30%	60%
The probability of infection with COVID-19	100/100,000	6/100,000
Which vaccine do you prefer?		

### Survey

During May 2021, the survey was conducted at vaccination sites in Qingdao, Shandong to everyone over the age of 18 and without cognitive impairment. Trained research students from Shandong University carried out the face-to-face interviews. The effective rate of survey completion was 95%. According to Orme (34), the minimum student sample size was 94, with our 1138 student observations surpassing the DCE minimum number. The survey was approved by Nanjing Medical University Ethics Committee and participants were informed about the purpose of the survey, that the data were for research purposes only and participants could withdraw from the survey at any stage of the interview.

### Data analysis

Our study deployed commonly used statistical models to measure utility trade-offs and preference heterogeneity. Conditional Logit Models (CLM) was used to measure individual vaccination preferences:


$$\begin{array}{l} U_{ijs}=\beta_{1}\text{effect}{\left(60\right)}_{\text{ijs}}+\beta_{2}\text{effect}{\left(85\right)}_{\text{ijs}}+\beta_{3}\text{sideeffect}{\left(10\right)}_{\text{ijs}}\\ \;+\beta_{4}\text{sideeffect}{\left(1\right)}_{\text{ijs}}+\beta_{5}\text{site}{\left(\text{secondlevel}\right)}_{\text{ijs}}\\ \;+\beta_{6}\text{site}{\left(\text{thirdlevel}\right)}_{\text{ijs}}+\beta_{7}\text{protection}{\left(1\text{yr}\right)}_{\text{ijs}}\\ \;+\beta_{8}\text{protection}{\left(2\text{yr}\right)}_{\text{ijs}}+\beta_{9}\text{acquaintances}{\left(60\right)}_{\text{ijs}}\\ \;+\beta_{10}\text{acquaintances}{\left(90\right)}_{\text{ijs}}+\beta_{11}\text{probinfected}{\left(6\right)}_{\text{ijs}}\\ \;+\beta_{12}\text{probinfected}{\left(100\right)}_{\text{ijs}}+{\text{ε}}_{\text{ijs}} \end{array}$$

Where *U*_*ijs*_ is the utility for individual *I* for scenario *j* (*j* = 1, 2) in the choice set s (*s* = 1, 2, 3). β are a fixed vector of parameters for each attribute level.

In addition, mixed logit model (MLM) was used to capture preference heterogeneity. Compared to the standard logit model, MLM allows for randomness across individuals by assuming that β follows a random distribution β ~*f*(β|θ). The MLM is specified as:


$$\begin{array}{l} U_{ijs}=\beta_{1}\text{effect}{\left(60\right)}_{\text{ijs}}+\beta_{2}\text{effect}{\left(85\right)}_{\text{ijs}}+\beta_{3}\text{sideeffect}{\left(10\right)}_{\text{ijs}}\\ \;+\beta_{4}\text{sideeffect}{\left(1\right)}_{\text{ijs}}+\beta_{5}\text{site}{\left(\text{secondlevel}\right)}_{\text{ijs}}\\ \;+\beta_{6}\text{site}{\left(\text{thirdlevel}\right)}_{\text{ijs}}+\beta_{7}\text{protection}{\left(1\text{yr}\right)}_{\text{ijs}}\\ \;+\beta_{8}\text{protection}{\left(2\text{yr}\right)}_{\text{ijs}}+\beta_{9}\text{acquaintances}{\left(60\right)}_{\text{ijs}}\\ \;+\beta_{10}\text{acquaintances}{\left(90\right)}_{\text{ijs}}+\beta_{11}\text{probinfected}{\left(6\right)}_{\text{ijs}}\\ \;+\beta_{12}\text{probinfected}{\left(100\right)}_{\text{ijs}}+{\text{ε}}_{\text{ijs}} \end{array}$$

Where *U*_*ijs*_ is the utility for individual *i* for scenario *j* (*j* = 1, 2) in the choice set s (*s* = 1, 2, 3) and β are a vector of random parameters for each individual and attribute level. We estimated the distribution of β for attribute levels to determine the existence of preference heterogeneity. To test model complexity and measure the goodness of fit of the CLM and MLM models, Akaike information criterion (AIC) and Bayesian information criterion (BIC) were calculated. While AIC has better predictive performance than BIC and is suitable for selecting the optimal model for predicting future observations, BIC is more efficient for choosing a correct model (35).

To better understand the underlying preference heterogeneity amongst students, sub-group analysis was performed by dividing the population into sub-groups based on shared characteristics such as risk tolerance and academic major. CLM was performed on each subgroup, and the results were compared cross sub-group to further analyse preference heterogeneity. Sub-group CLM specifications were estimated using the equations:


$$\begin{array}{l} U_{\text{ijrs}}=\beta_{1\text{r}}\text{effect}{\left(60\right)}_{\text{ijrs}}+\beta_{2\text{r}}\text{effect}{\left(85\right)}_{\text{ijrs}}+\beta_{3\text{r}}\text{sideeffect}{\left(10\right)}_{\text{ijrs}}\\ \;+\beta_{4\text{r}}\text{sideeffect}{\left(1\right)}_{\text{ijrs}}+\beta_{5\text{r}}\text{site}{\left(\text{secondlevel}\right)}_{\text{ijrs}}\\ \;+\beta_{6r}\text{site}{\left(\text{thirdlevel}\right)}_{\text{ijrs}}+\beta_{7r}\text{protection}{\left(1\text{yr}\right)}_{\text{ijrs}}\\ \;+\beta_{8\text{r}}\text{protection}{\left(2\text{yr}\right)}_{\text{ijrs}}+\beta_{9\text{r}}\text{acquaintances}{\left(60\right)}_{\text{ijrs}}\\ \;+\beta_{10\text{r}}\text{acquaintances}{\left(90\right)}_{\text{ijrs}}+\beta_{11\text{r}}\text{probinfected}{\left(6\right)}_{\text{ijrs}}\\ \;+\beta_{12r}\text{probinfected}{\left(100\right)}_{\text{ijrs}}+{\text{ε}}_{\text{ijrs}} \end{array}$$

Where *U*_*ijrs*_ is the utility for individual *i* for scenario *j* (*j* = 1, 2) in the choice set s (*s* = 1, 2, 3) and sub-group *r* (*r* = 1, 2). β are a fixed vector of parameters for each attribute level.

## Results

### Sample characteristics

Of the 1,138 students sampled, 515 (45.3%) were male; 814 (71.5%) were from urban regions; 478 (42%) students studied humanities/social sciences and 660 (58%) students majored in other fields (science, engineering, agriculture and medicine). For risk tolerances, 872 (76.6%) students reported as being risk adverse, 132 (11.6%) were unsure and 134 (11.8%) were risk tolerant; 16 (1.4%) reported low trust in government, 45 (4%) students were unsure, and 1,054 (92.6%) viewed the government as being trustworthy. Other characteristics such as age, family household income and marital status were also collected, and the detailed characteristics of the student sample are shown in Table 3.

**Table 3:** Characteristics of the student sample (*n* = 1,138).

**Characteristics**	***n***	**%**
**Sex**		
Male	515	45.3
Female	623	54.7
**Marital status**		
Married	18	1.6
Unmarried/widowed/divorced	1,120	98.4
**Residence**		
Urban area	814	71.5
Rural area	324	28.5
**Household yearly income**		
Low income level	174	15.3
Medium income level	819	72.0
High income level	145	12.7
**Major**		
Humanities/social science	478	42.0
Non-humanities/social science	660	58.0
**Trust in government**		
Mistrust	16	1.4
Unsure	45	4.0
Trust	1,054	92.6
**Risk preference**		
Risk adverse	872	76.6
Unsure	132	11.6
Risk tolerant	134	11.8

### Estimation of parameters

Table 4 presents the results of the CLM. When compared to the reference levels, vaccine effectiveness (60%), vaccine related side-effects, vaccination sites, duration of protection, percentage of acquaintances vaccinated and the probability of infection were all statistically significant. Vaccine effectiveness (85%) was not statistically significant at the 5% level. Our results show that students preferred vaccines with fewer side-effects, administered through third level county hospitals and with at least 1 year duration of protection. In addition, students were more inclined to vaccinate when they perceive a higher risk of infection for themselves or their acquaintances. AIC and BIC results are also reported and model comparisons were conducted based on these goodness-of-fit statistics. Notably, student's vaccination utility decreased when the percentage of acquaintances vaccinated increases from the 30% level to the 60% level. This suggests that student vaccination preferences are dependent upon vaccination choices of acquaintances around them. Overall high vaccination rates entice free riding issues, where students are demotivated to vaccinate because they can enjoy the vaccination benefits of others (36). Surprisingly, this phenomenon is not evident for the 90% acquaintances vaccinated level, perhaps due to higher social conformity pressures.

**Table 4:** Conditional logit model of respondent preferences.

**Attribute**	**β**	**SE**	***p*-Values**	**95% CI**
**Vaccine effectiveness (reference = 40%)**				
60%	0.423	0.036	0.000	0.351, 0.494
85%	0.806	0.041	0.000	0.727, 0.886
**Vaccine-related side effects (reference = 50/100,000)**				
10/100,000	0.251	0.035	0.000	0.182, 0.320
1/100,000	0.432	0.037	0.000	0.358, 0.507
**Vaccination sites (reference = Level 1)**				
Level 2	0.141	0.037	0.000	0.067, 0.214
Level 3	−0.067	0.036	0.063	−0.138, 0.004
**Duration of vaccine protection (reference = 6 months)**				
1 year	0.245	0.037	0.000	0.173, 0.316
More than 2 years	0.350	0.036	0.000	0.279, 0.421
**Acquaintances vaccinated (reference = 30%)**				
60%	0.031	0.037	0.409	−0.042, 0.103
90%	0.093	0.037	0.011	0.021, 0.165
**The probability of respondents/acquaintances infected (reference = 1/100,000)**				
6/100,000	0.221	0.037	0.000	0.148, 0.293
100/100,000	0.346	0.036	0.000	0.274, 0.417
Model fit				

Prob > chi^2^ = 0.000, Pseudo R^2^ = 0.1024, LR chi^2^(13) = 1,292.28, AIC = 1,1352.54, BIC = 1,1446.26.

The results of the mixed logit model are presented in Table 5. Similar to the fixed effects model, we observe that all attributes, except for the 85% vaccine effectiveness level, were statistically significant at the 5% level. Individual preferences under the MLM are in line with our CLM results. In particular, vaccine side-effects were the predominant attribute affecting student decision making, followed by duration of protection and perception of risk. Vaccination sites, vaccine effectiveness and percentage of acquaintances vaccinated were less important attributes. More importantly, when assuming that β follows a random distribution β ~*f*(β|θ) under the MLM, we observe preference heterogeneity amongst students for vaccine side-effects, vaccination sites and duration of protection (2 years).

**Table 5:** Mixed logistic regression models of student preferences.

**Variables**	**Coefficients**	**SE**	***p*-Values**	**95% CI**
**Mean**				
Vaccine effectiveness (reference = 40%)				
60%	−0.240	0.041	0.000	−0.320, −0.160
85%	−0.082	0.0481	0.087	−0.177, 0.012
Vaccine-related side effects (reference = 50/100,000)				
10/100,000	0.695	0.042	0.000	0.612, 0.778
1/100,000	1.027	0.050	0.000	0.928, 1.126
Vaccination sites (reference = level 1)
Level 2	0.131	0.042	0.002	0.048, 0.214
Level 3	0.166	0.045	0.000	0.078, 0.255
Duration of vaccine protection (reference = 6 months)				
One year	0.488	0.042	0.000	0.405, 0.570
More than 2 years	0.311	0.042	0.000	0.228, 0.393
Acquaintances vaccinated (reference = 30%)				
60%	−0.381	0.041	0.000	−0.461, −0.300
90%	0.167	0.042	0.000	0.085, 0.249
The probability of respondents/acquaintances infected (reference = 1/100,000)				
6/100,000	0.318	0.042	0.000	0.236, 0.400
100/100,000	0.590	0.043	0.000	0.505, 0.675
**SE**				
Vaccine effectiveness (reference = 40%)				
60%	0.012	0.108	0.914	−0.201 0.224
85%	0.412	0.090	0.000	0.234, 0.589
Vaccine related side-effects (reference = 50/100,000)				
10/100,000	0.417	0.083	0.000	0.254, 0.580
1/100000	0.015	0.324	0.963	−0.620, 0.650
Vaccination sites (reference = level 1 village community health station)				
Level 2	0.510	0.078	0.000	0.357, 0.663
Level 3	0.827	0.071	0.000	0.687, 0.966
Duration of vaccine protection (reference = 6 months)				
One year	0.202	0.169	0.232	−0.130, 0.534
More than 2 years	0.518	0.073	0.000	0.375, 0.662
Acquaintances vaccinated (reference = 30%)				
60%	0.173	0.186	0.353	−0.192, 0.537
90%	−0.002	0.088	0.978	−0.174, 0.169
The probability of respondents/acquaintances infected (reference = 1/100,000)				
6/100,000	−0.027	0.117	0.817	−0.257, 0.203
100/100,000	0.502	0.080	0.000	0.346, 0.659

To better understand the underlying preference heterogeneities, we performed subgroup analysis by dividing our cohort according to different student characteristics. We sub-divided our student population based on the following two key characteristics: student's major (humanities/social sciences or non-humanities/social sciences) and risk tolerance (risk adverse or risk tolerant). Based on our defined sub-groups, we performed CLM for each subgroup and compared the cross-group results to further investigate existing preference heterogeneity. Sub-group analysis by student major in Table 6 shows that there are no observable preference heterogeneities. Similar to the whole student sample, both humanities/social science majors and non-humanities/social science majors preferred vaccines which were safer, administered in third level county hospitals and had 1 year of protection. As shown in Table 7 for the sub-group analysis based on student's risk tolerance, the likelihood of vaccination increased with an increase in perception of risk, and vaccination utility decreased when percentage of acquaintances increased from 30 to 60%, revealing a free rider problem where individual vaccination preferences are affected by the vaccination decisions of acquaintances around them. In particular, high percentages of acquaintances vaccinated may demotivate students to vaccinate, resulting in low vaccination utility, as they can benefit from herd immunity while avoiding the cost of vaccination themselves (36). In addition, when compared to the baseline, vaccine effectiveness at the 60% level surprisingly reduced both cohort's utility, and the 85% effective level was not statistically significant for both cohorts.

**Table 6:** Sub-group analysis for student majors.

**Attribute**	**β**	**SE**	***p*-Values**	**β**	**SE**	***p*-Values**
**Sub-group**	**Social sciences**			**Non-social sciences**		
**Vaccine effectiveness (reference =40%)**						
60%	−0.151	0.048	0.001	−0.137	0.041	0.001
85%	0.052	0.051	0.311	−0.0270	0.044	0.541
**Vaccine related side-effects (reference = 50/100,000)**						
10/100,000	0.538	0.049	0.000	0.596	0.043	0.000
1/100,000	0.772	0.052	0.000	0.901	0.044	0.000
**Vaccination sites (reference = level 1 village community health station)**						
Level 2	0.104	0.051	0.040	0.168	0.048	0.000
Level 3	0.144	0.047	0.002	0.187	0.041	0.000
**Duration of vaccine protection (reference = 6 months)**						
One year	0.387	0.050	0.000	0.404	0.044	0.000
More than 2 years	0.166	0.048	0.001	0.231	0.042	0.000
**Acquaintances vaccinated (reference = 30%)**						
60%	−0.282	0.049	0.000	−0.385	0.043	0.000
90%	0.100	0.048	0.038	0.096	0.042	0.021
**The probability of respondents/acquaintances infected (reference = 1/100,000)**						
6/100,000	0.245	0.050	0.000	0.281	0.044	0.000
100/100,000	0.476	0.047	0.000	0.503	0.040	0.000

**Table 7:** Sub-group analysis for student risk preferences.

**Attribute**	**β**	**SE**	***p*-Values**	**β**	**SE**	***p*-Values**
**Sub-group**	**Risk adverse**			**Risk tolerant**		
**Vaccine effectiveness (reference=40%)**						
60%	−0.181	0.036	0.000	0.140	0.090	0.117
85%	−0.039	0.038	0.303	0.296	0.098	0.003
**Vaccine related side-effects (reference=50/100,000)**						
10/100,000	0.621	0.037	0.000	0.357	0.092	0.000
1/100,000	0.881	0.039	0.000	0.568	0.098	0.000
**Vaccination sites (reference = level 1 village community health station)**						
Level 2	0.125	0.038	0.001	0.318	0.098	0.001
Level 3	0.218	0.035	0.000	0.168	0.090	0.063
**Duration of vaccine protection (reference = 6 months)**						
One year	0.395	0.037	0.000	0.396	0.095	0.000
more than 2 years	0.234	0.036	0.000	0.050	0.092	0.591
**Acquaintances vaccinated (reference = 30%)**						
60%	−0.303	0.037	0.000	−0.443	0.098	0.000
90%	0.133	0.036	0.000	0.014	0.091	0.875
**The probability of respondents/acquaintances infected (reference = 1/100,000)**						
6/100,000	0.248	0.038	0.000	0.437	0.097	0.000
100/100,000	0.459	0.035	0.000	0.587	0.089	0.000

We also observed preference heterogeneity for vaccine effectiveness, vaccination sites, duration of protection and acquaintances vaccinated when dividing our student population by risk tolerance. Risk tolerant students preferred township community health station sites over higher level three county hospital vaccination sites, and exhibited no significant preference between vaccines with 6 months protection or 2 years of protection. By contrast, students who were risk adverse preferred to be administered vaccines at third level county hospitals and had clear preferences for vaccines with longer duration of protection compared to the baseline 6 month of protection. Having high percentages (90%) of acquaintances vaccinated did not statistically effect risk tolerant students' vaccination preferences, but did have a positive effect on risk adverse students' vaccination uptake.

## Discussion

Comprising both intrinsic and extrinsic attributes, our study is the first DCE study to investigate mainland Chinese university student vaccination preferences. We found that students preferred vaccines which had fewer side-effects, had longer duration of protection and administered through second or third level health facilities. In addition, the perception of higher risks of infection generally lead to higher vaccine uptake. These results are broadly consistent with previous DCE studies on the general public (16–18). Notably, students exhibited vaccination free riding issues at the 60% level, which had not been reported by any other DCE studies, and free riders were a significant problem for the risk adverse student subgroup. From the β coefficients of the CLM, we observe that safety, duration of protection and perception of risk were the three most influential attributes. When compared against studies conducted on the general public before the implementation of China's 2021 vaccination program, we observe that duration of protection has become more influential relative to intrinsic vaccine attributes such as safety and effectiveness (17). This is consistent with findings of studies conducted on the general public after China's 2021 vaccination program (18). However, the Hong Kong based student surveys reported that duration of protection was the least influential attribute (29, 30), perhaps due to the different vaccination contextual backgrounds, such as low trust in government (37) and varying policy settings, which makes inferring the Hong Kong results to mainland Chinese vaccination policies potentially misleading.

Mixed logit models demonstrated clear preference heterogeneity for vaccine intrinsic attributes among students. To better analyze the underlying preference disparities, we conducted sub-group analysis based on student majors (humanities/social science or non-humanities/social science) and risk tolerance (risk tolerant or risk adverse). Our results support previous online survey studies which reported that there are no statistically significant relationships between student majors and vaccine preferences (26). Given our academic major data are social science and non-social science, our results do not contribute to the prior findings that medical and nursing students had higher vaccine willingness than other students due to higher knowledge levels and better perception of vaccines (28, 38). When stratifying the population by risk tolerance, we observed that risk tolerant students exhibited preferences distinct to the risk adverse cohort. In particular, risk tolerant students showed no trade-off in utility between 6 months and 2 years of vaccine protection. In contrast to the whole student population, and consistent with previous findings for the general public (18), risk tolerant students preferred to be administered vaccines at second level medical centers over third level county hospitals. One possible explanation is that the need for convenience triumphs over perceived vaccination administration risks at second level health facilities (18).

There are some limitations of our study. The sampling of this study occurred concurrently with the DCE survey for the Qingdao general public (18). Compared to online surveys, our study was a DCE conducted face-to-face, but due to the COVID-19 travel and social restrictions, our data was limited to one city in China. Further student DCE studies should be undertaken in other cities. In addition, our sampling at vaccination centers may leave out those who are vaccine hesitant and future studies should focus on determining factors that can motivate these populations. While acknowledging face-to-face interviews may risk social desirability bias, especially for distrust of government or risk aversion items, our trained interviewers and privacy protocols attenuated this problem. We collected limited information with regard to student majors, and future studies should collect more detailed data on student majors.

## Conclusion

Students have been identified as a major risk group during the 2022 outbreaks of COVID-19 in China. Our DCE study investigated Qingdao university student vaccination preferences for both intrinsic and extrinsic attributes. We found that most students exhibited preferences similar to the general public, but risk tolerant students exhibited clear preference heterogeneity for vaccination sites and duration of protection. In addition, our results revealed free riding issues, where high vaccination rates among acquaintances reducing the intention to vaccinate, especially for risk adverse subgroup students.

Our results inform universities and local governments across China on the implementation of their vaccination programs, especially for students. Understanding the existence of vaccination free riding and preference discrepancies between student sub-groups guide local governments to targeted COVID-19 vaccination campaigns for younger people and subsets of students. We recommend on-campus vaccination education programs to provide targeted messaging for different student cohorts, especially to address the risk of free riding. Such targeted programs are urgent with the return to on-campus teaching and to attenuate the risk of COVID-19 resurgence.

## Data availability statement

The raw data supporting the conclusions of this article will be made available by the authors, without undue reservation.

## Ethics statement

The studies involving human participants were reviewed and approved by Nanjing Medical University Ethics Committee. The patients/participants provided their written informed consent to participate in this study.

## Author contributions

SW and AL contributed to the conception, design, and methodology of the manuscript. SW wrote the initial draft of the preparation, conducted the formal analysis of the data, including data curation, and visualization. EM, SN, AL, and TW contributed to the manuscript revision and supervision. AL contributed to the project's administration, data collection, and funding acquisition. All authors read and approved the final version of the manuscript.

## Funding

This work was supported by the National Natural Science Foundation of China (grant number 72004117) and China Postdoctoral Science Foundation (grant number 2019M662392).

## Conflict of interest

The authors declare that the research was conducted in the absence of any commercial or financial relationships that could be construed as a potential conflict of interest.

## Publisher's note

All claims expressed in this article are solely those of the authors and do not necessarily represent those of their affiliated organizations, or those of the publisher, the editors and the reviewers. Any product that may be evaluated in this article, or claim that may be made by its manufacturer, is not guaranteed or endorsed by the publisher.
